# Maternal and neonatal outcomes of singleton versus twin pregnancies complicated by gestational diabetes mellitus: A systematic review and meta-analysis

**DOI:** 10.1371/journal.pone.0280754

**Published:** 2023-01-25

**Authors:** Fengming Tu, Aimei Fei

**Affiliations:** Huzhou Maternity&Child Health Care Hospital, Huzhou, Zhejiang Province, China; University of Abuja Teaching Hospital, NIGERIA

## Abstract

**Background:**

There is limited evidence exploring the maternal and neonatal complications of gestational diabetes mellitus (GDM) following singleton or twin pregnancies. Further, there have been no reviews completed examining the possible risk factors associated with GDM in singleton compared to twin pregnancies. This study assesses the impact of GDM in singleton and twin pregnancies on maternal and neonatal outcomes.

**Methods:**

From 1954 to December 2021, a thorough literature search was conducted in the EMBASE, Cochrane, MEDLINE, ScienceDirect, and Google Scholar databases and search engines. The risk of bias was calculated using the Newcastle Ottawa (NO) scale. A random-effects model was applied and interpreted as pooled odds ratio (OR) with 95% confidence intervals (CI).

**Results:**

Eight studies satisfied the inclusion criteria, with the quality of most studies being good to satisfactory. The risk of caesarean section (pooled OR = 0.32; 95%CI: 0.22 to 0.46), small-for-gestational age (SGA) neonates (pooled OR = 0.40; 95%CI: 0.19 to 0.84), preterm delivery (pooled OR = 0.07; 95%CI: 0.06 to 0.09), respiratory morbidity (pooled OR = 0.26; 95%CI: 0.19 to 0.37), neonatal hyperbilirubinemia (pooled OR = 0.19; 95%CI: 0.10 to 0.40), and NICU admission (pooled OR = 0.18; 95%CI: 0.14 to 0.25) was significantly lower in singleton pregnancies with GDM than in twin pregnancies with GDM.

**Conclusion:**

Maternal outcomes like caesarean section and neonatal outcomes like SGA neonates, preterm delivery, respiratory morbidity, hyperbilirubinemia, and NICU admission were significantly greater in twin pregnancies with GDM. It is important for clinicians and policymakers to focus intervention strategies on twin pregnancies with GDM.

## Introduction

Gestational diabetes mellitus (GDM), which is defined as glucose intolerance that develops and is first recognized during pregnancy, is one of the most common complications in pregnant women. The burden of GDM has been increasing globally, over the past decade [1]. There has been a concurrent rise in the rate of twin pregnancy [2–4]. The risk factors associated with GDM and twin pregnancy are shared, such as high body mass index (BMI) and advancing maternal age [5]. GDM increases the risk of preterm delivery and a twin pregnancy with GDM is associated with a higher incidence of adverse pregnancy outcomes, such as gestational hypertension, pre-eclampsia, premature rupture of membranes, and adverse neonatal outcomes, such as small for gestational age (SGA) offspring, low birth injuries, hypoglycaemia, hypothermia, respiratory morbidity and neonatal intensive care unit (NICU) admission [6–9]. Of note, a singleton pregnancy with GDM has its own set of complications such as respiratory morbidity, macrosomia, hypoglycaemia and shoulder dystocia [10, 11]. However, the limited literature examining the link between GDM in singleton and twin pregnancies and maternal and neonatal complications is conflicting [12–14]. The available evidence is limited by small sample sizes or lack of a control group of singleton pregnancies, thereby removing the possibility of assessing the impact of plurality on the clinical outcomes due to GDM [12–14]. To date, there are no studies that attempted to summarize data on the difference in outcomes between singleton and twin pregnancies in patients with GDM. Thus, the aim of the current review and meta-analysis was to summarize data from studies that examine the difference in maternal and neonatal outcomes between singleton and twin pregnancies in women with GDM.

## Methods

### Eligibility criteria

#### Study design

Observational studies that meet the criteria for inclusion (cross-sectional, cohort, case-control, etc.) were included. Conference abstracts, case studies, case reports, and grey literature were excluded whereas full-text papers were included. This study was registered in PROSPERO (CRD42022301059).

#### Study participants

Studies containing women diagnosed with GDM were included, while studies with pregnant women with pre-existing diabetes were excluded.

#### Exposure

Studies evaluating the difference in maternal and neonatal outcomes in singleton and twin pregnancies complicated by GDM.

#### Outcome

*Maternal outcomes*. Gestational hypertension, pre-eclampsia, caesarean section.

*Neonatal outcomes*. Preterm delivery, small for gestational age (SGA) neonates, low birth weight, hypoglycaemia, hyperbilirubinemia or jaundice, respiratory morbidity, and NICU admission.

### Search strategy

EMBASE, Cochrane Library, MEDLINE, and search engines like Google Scholar and ScienceDirect were used in the literature search (**S1 Appendix**). The search was conducted using free-text phrases and medical subject headings (MeSH). The time period (January 1954 to December 2021), language (English only), and design filters were all used during the final search, which was carried out using appropriate Boolean operators ("AND" & "OR") and observational study filter. The relevant articles’ references were checked for any new publications.

## Study selection process

1. The titles and abstracts of the papers that had been identified were examined by two separate researchers (FT & AF). If a study met the inclusion requirements, the full text of the study was retrieved.
2. The same group of investigators (FT & AF) then conducted a screening of the retrieved full texts and evaluated them in light of the inclusion criteria. Studies that met the criteria were included, and the reasons for excluding the others were noted.
3. Any discrepancies were discussed with the second investigator in order to be rectified (AF). The study was reported in accordance with the "Preferred Reporting Items for Systematic Reviews and Meta-Analyses (PRISMA) 2020 checklist" [15].

### Data extraction process

Data extracted for the review consisted of the following information: Authors, the title of the study, the year it was published, the study period, the study design, the setting, the country or region, the total sample size, the details of the outcome assessment, the average age, and the primary and secondary outcomes in each group. The first author (FT) entered the data, while the second author (AF) double-checked the accuracy of entry.

### Risk of bias assessment

The Newcastle Ottawa (NO) scale for observational studies was used to assess the likelihood of bias independently by two investigators (FT and AF). The domains it covers are Selection (four stars), Comparability (two stars), and Outcome (two stars). The final score can vary from 0 to 8 stars; studies with a score of 7–8 stars are considered to be of "good," "satisfactory," or "unsatisfactory" quality, respectively [16].

### Statistical analysis

STATA version 14.2 was used for the statistical analysis (StataCorp, CollegeStation, TX, USA). The data were presented as a pooled odds ratio (OR) because the primary outcomes were binary, and the number of events and sample size within each group were entered. The methodological heterogeneity was taken into account using a random effects model with inverse-variance.

The chi-square test and the I^2^ statistic were used to assess heterogeneity [17]. The pooled estimate and study-specific estimate were graphically represented using a forest plot, and the robustness of the pooled estimate was assessed using a sensitivity analysis. Due to the final inclusion of less than 10 studies, a meta-regression or assessment of publication bias could not be done (8 studies).

## Results

### Study selection

**Fig 1:**
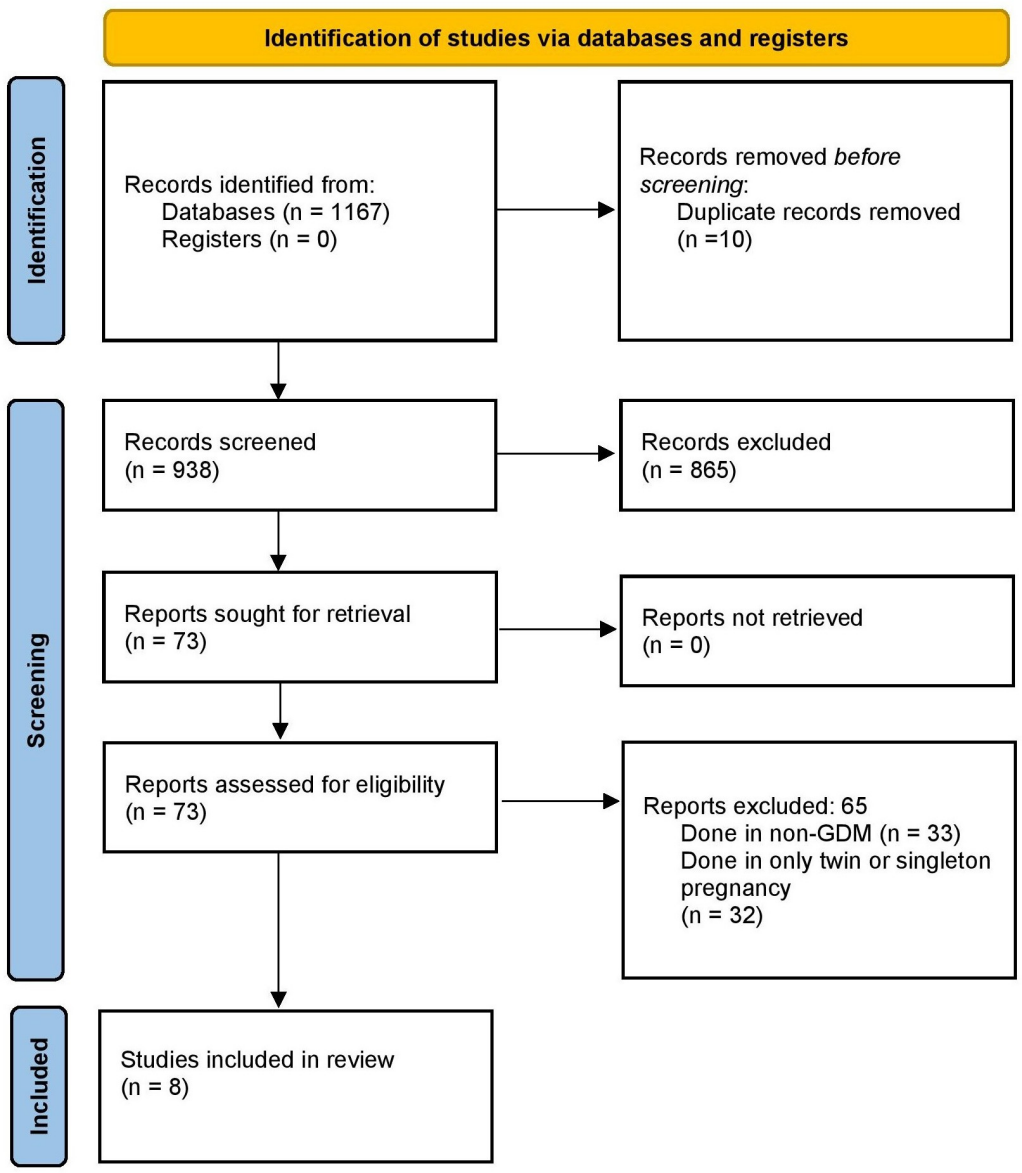
PRISMA flowchart.

In total, 1,167 records were identified through literature search. Of them, 73 pertinent study full texts were acquired. By looking through the references of the obtained full texts during primary screening, two additional publications were found. The final decision was made to include 8 studies with 41,548 participants after the final screening against eligibility criteria (Fig 1) [12–14, 18–22].

### Characteristics of the included studies

Included studies were all retrospective in nature. Most were conducted in Canada (3 studies) followed by Spain (2 studies). The sample sizes varied from 184 to 18,942. The average age of women with a singleton pregnancy complicated with GDM ranged from 32 to 35.5 years and women with a twin pregnancy and GDM from 31.9 to 35.8 years. Seven of the studies reported on the SGA outcome, 6 studies each on pre-eclampsia and caesarean section outcomes, 5 studies on preterm birth, and 4 studies each on respiratory morbidity, hypoglycaemia, hyperbilirubinemia, and NICU admission, and 3 studies on gestational hypertension (**Table 1**). The majority (5 out of 6) of the studies were of good to satisfactory quality (**Table 2**).

**Table 1:** Characteristics of the included studies (N = 8).

Author and year	Country	Study design	Sample size in singleton GDM	Sample size in twin GDM	Study participants	Mean age
Ashwal 2021	Canada	Retrospective cohort	1893	180	All women with a singleton or twin pregnancy (with outcome reported separately for GDM patients with singleton or twin pregnancy) who were followed up at a single tertiary referral center (Sunnybrook Health Sciences Center, Toronto, Ontario, Canada) between January 1, 2011, and April 31, 2020	S = 34.6 T = 35.1
Guillén-Sacoto 2017	Spain	Retrospective observational	122	62	GDM pregnancies, women were followed throughout their pregnancy, then underwent postpartum examination between January 1991 and December 2015.	S = 35.5 T = 35.8
Guillén-Sacoto 2018	Spain	Retrospective observational	240	120	Pregnant women with GDM: 120 pregnancies twins and 240 singleton pregnancies as controls	S = 35.1 T = 35.3
Hiersch 2019	Canada	Retrospective cohort	16731	649	All twin and singleton live births in Ontario, Canada, 2012–2016 with pregnancy outcomes compared between women with vs without gestational diabetes mellitus, analysed separately for twin and singleton births	S = 33.1 T = 34
Lai 2015	Canada	Retrospective analysis	18137	805	Singleton and twin GDM pregnancies resulting in live/still births in Alberta	NR
Sheehan 2019	Australia	Retrospective cohort	1370	39	All women who delivered at the Royal Women’s Hospital, Melbourne, between January 2016 and December 2017 with outcome reported separately between GDM singleton and GDM twin pregnancy	NR
Weiner 2018	Israel	Retrospective analysis	228	114	All deliveries of singleton and twin pregnancies complicated by GDM at a single university-affiliated hospital between 1/2008-10/2016	S = 32.3 T = 31.9
Xue 2019	China	Retrospective analysis	572	286	Twin and singleton pregnancies complicated by GDM, which delivered in Peking University First Hospital from January 1st, 2012—December 31st, 2017	S = 32 T = 33

S-Singleton pregnancy; T-Twin pregnancy; NR-Not reported

**Table 2:** Quality assessment of the included studies (N = 8).

S.N.	Author and year	Representativeness	Sample size justification	Non-response	Ascertainment of exposure	Control for confounding	Assessment of outcome	Statistical tests	Overall Quality
1.	Ashwal 2021	*	0 star	0 star	*	**	*	*	Satisfactory
2.	Guillén-Sacoto 2017	*	*	*	*	*	*	*	Good
3.	Guillén-Sacoto 2018	*	0 star	*	*	*	*	*	Good
4.	Hiersch 2019	*	*	0 star	*	*	*	*	Good
5.	Lai 2015	0 star	0 star	0 star	*	**	*	*	Satisfactory
6.	Sheehan 2019	*	0 star	0 star	*	**	*	*	Satisfactory
7.	Weiner 2018	*	*	*	*	*	*	*	Good
8.	Xue 2019	*	0 star	*	*	**	*	*	Good

### Maternal outcomes

#### Gestational hypertension

**Fig 2:**
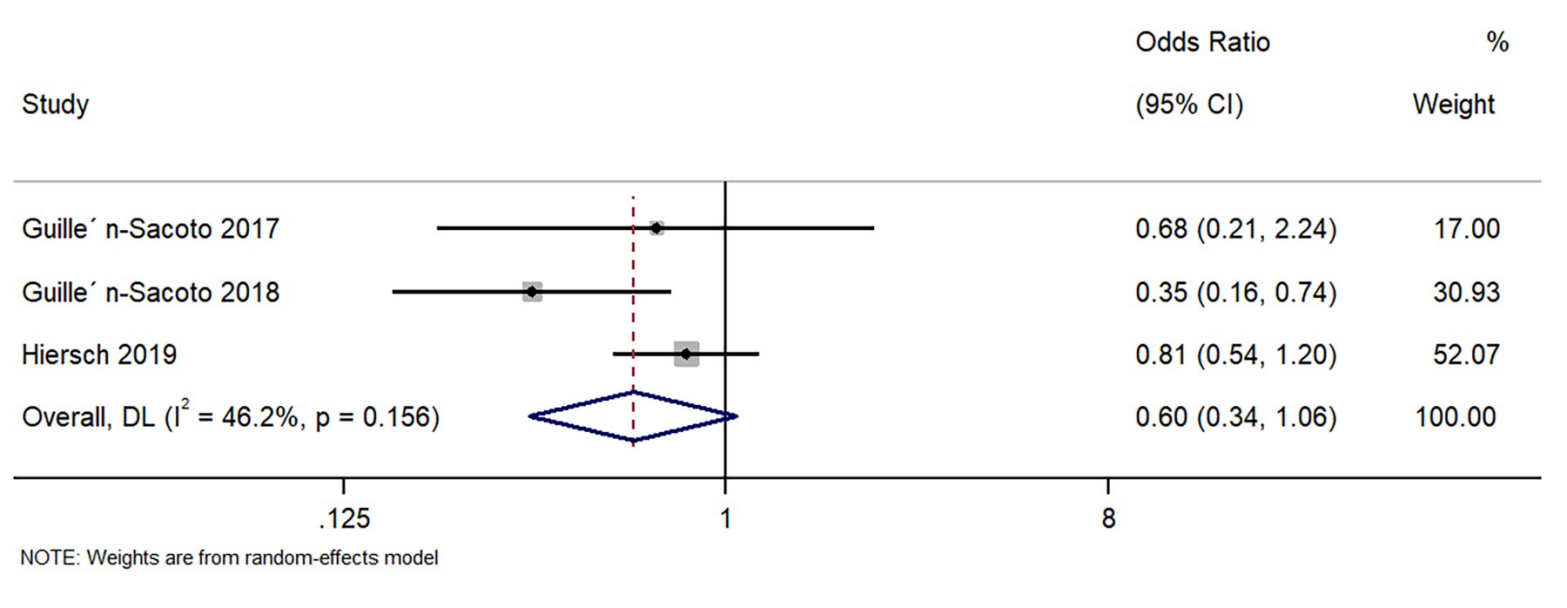
Forest plot showing the difference in gestational hypertension between women with a singleton pregnancy or twin pregnancy and GDM. NOTE: Weights are from random-effects model.

Three studies described the difference in gestational hypertension between singleton GDM and twin GDM pregnancies. The pooled OR was 0.60 (95%CI: 0.34 to 1.06; I^2^ = 46.2%), indicating a similar incidence of gestational hypertension in women with singleton GDM and twin GDM pregnancies(p = 0.08) (Fig 2).

#### Pre-eclampsia

**Fig 3:**
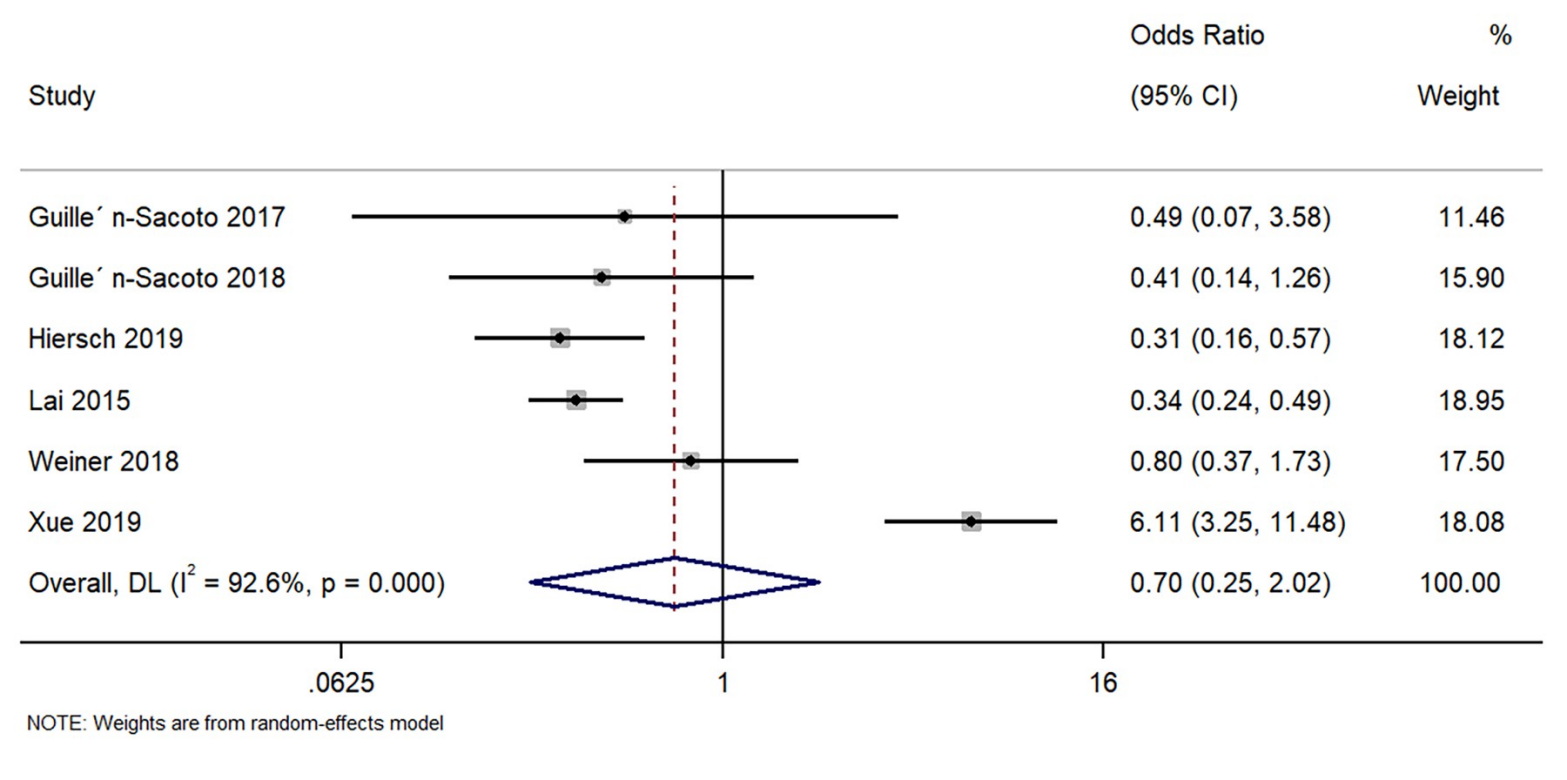
Forest plot showing the difference in pre-eclampsia between women with a singleton pregnancy or twin pregnancy and GDM. NOTE: Weights are from random-effects model.

Six studies described the difference in pre-eclampsia between singleton GDM and twin GDM pregnancies. The pooled OR was 0.70 (95%CI: 0.25 to 2.02; I^2^ = 92.6%), suggesting no difference in the rate of pre-eclampsia between singleton GDM and twin GDM pregnancies (p = 0.51) (Fig 3).

### Caesarean section

**Fig 4:**
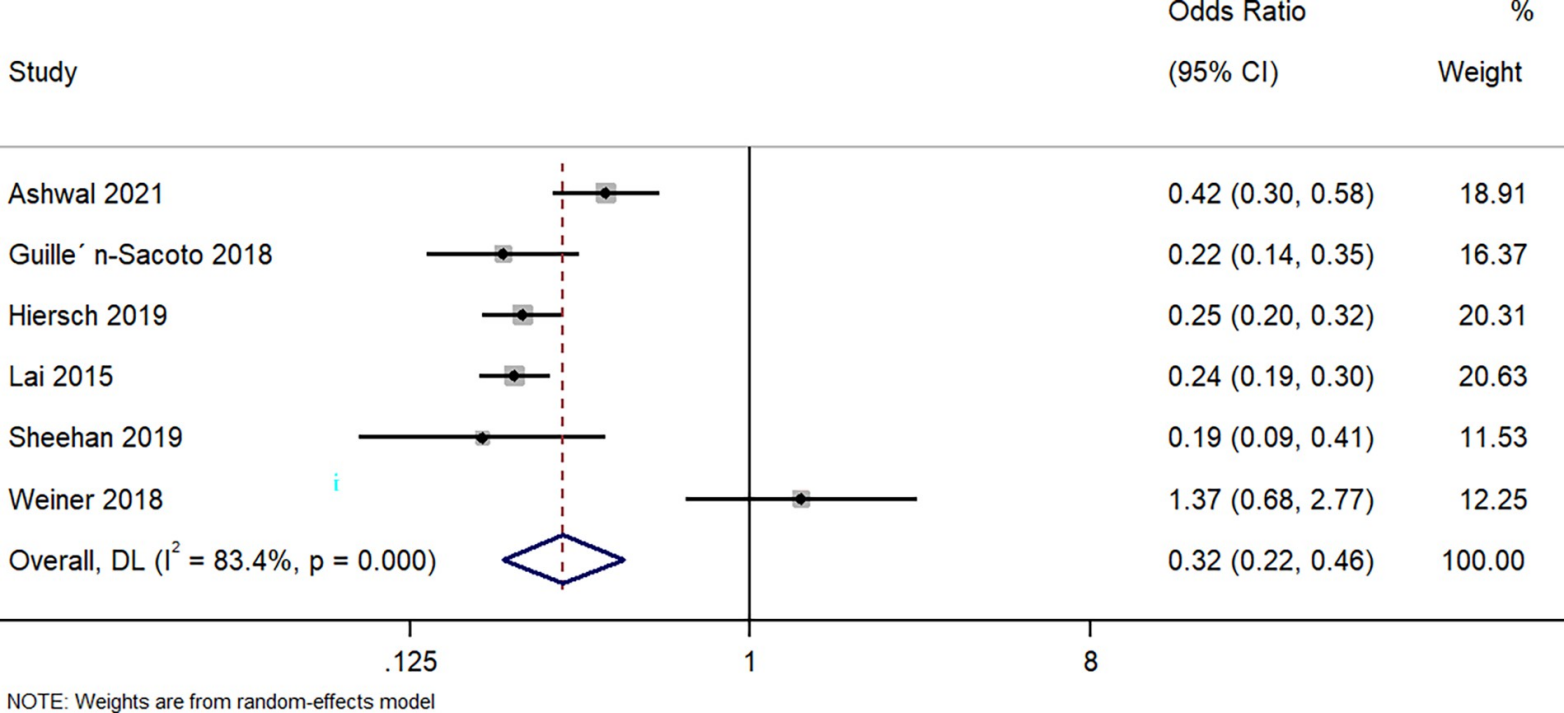
Forest plot showing the difference in caesarean section between women with a singleton pregnancy or twin pregnancy and GDM. NOTE: Weights are from random-effects model.

Six studies described the difference in the caesarean section between singleton GDM and twin GDM pregnancies. The pooled OR was 0.32 (95%CI: 0.22 to 0.46; I^2^ = 83.4%), indicating that a singleton pregnancy complicated with GDM correlates with a markedly (68%) lower risk of the caesarean section when compared to a twin GDM pregnancy (p<0.001) (Fig 4).

### Neonatal outcomes

#### Small for gestational age neonates

**Fig 5:**
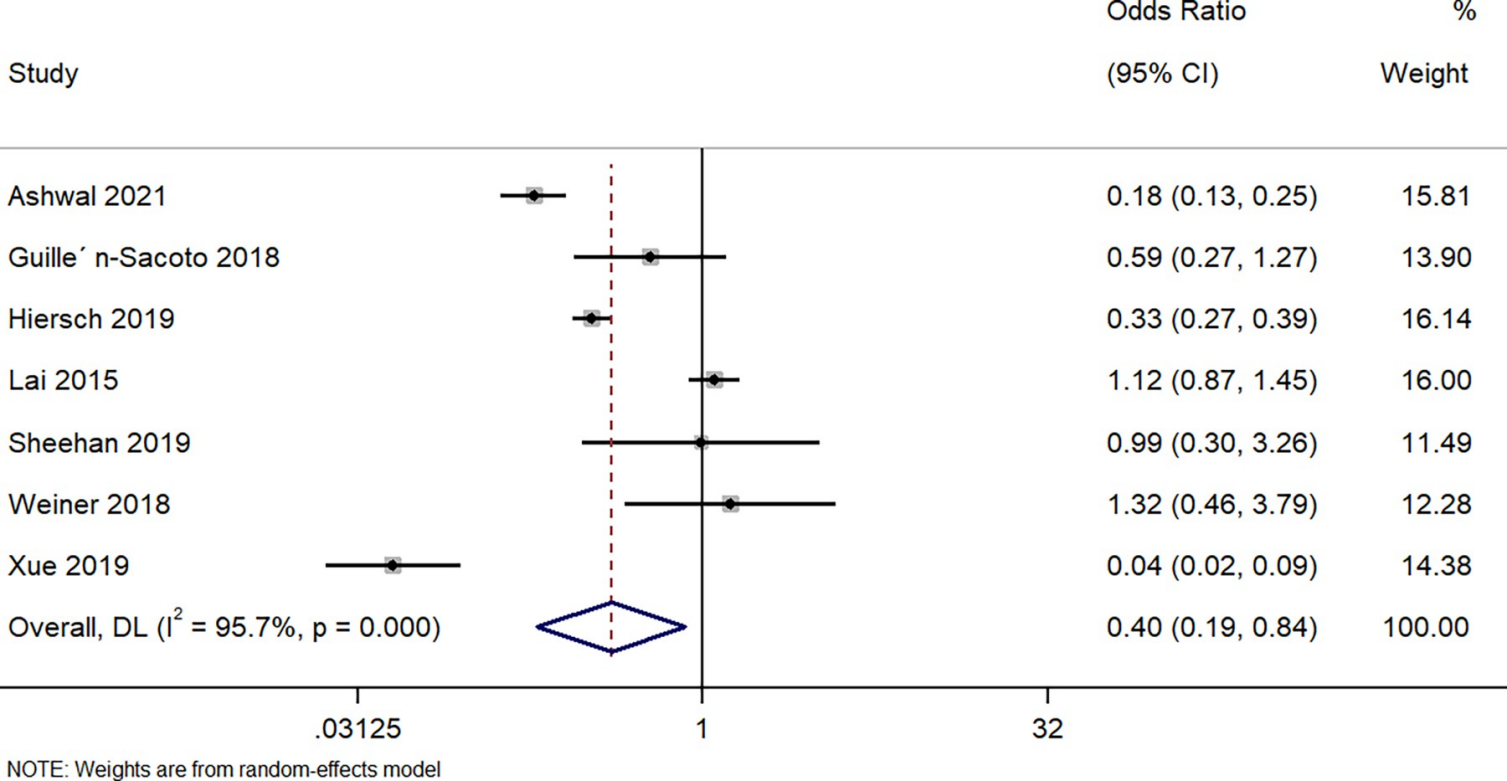
Forest plot showing the difference in small for gestational age neonates between women with a singleton pregnancy or twin pregnancy and GDM. NOTE: Weights are from random-effects model.

Seven studies described the difference in delivering an SGA neonate between singleton GDM and twin GDM pregnancies. The pooled OR was 0.40 (95%CI: 0.19 to 0.84; I^2^ = 95.7%), indicating that the women with a singleton pregnancy and GDM had a significantly (60%, p = 0.01) lower risk of delivering small for gestational age offspring than women with a twin pregnancy and GDM (Fig 5).

#### Preterm delivery

**Fig 6:**
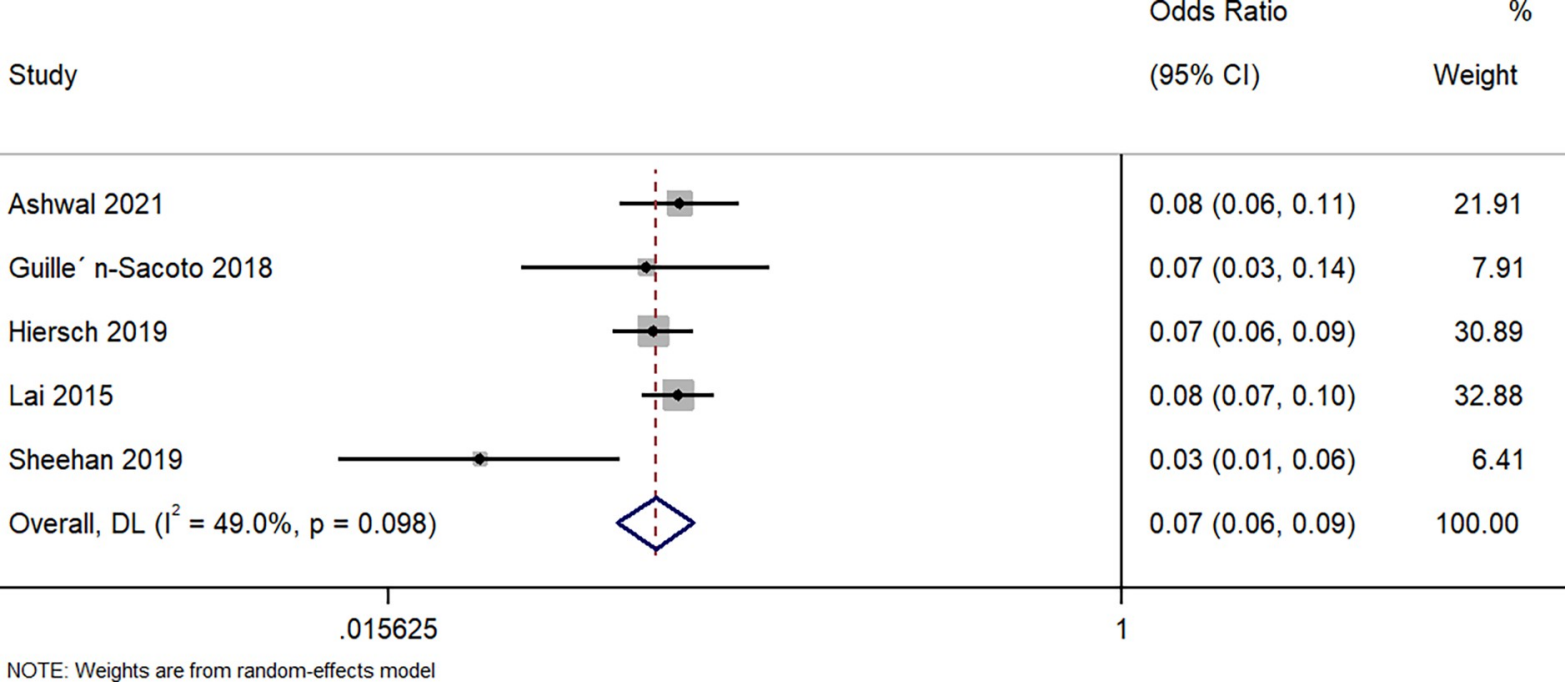
Forest plot showing the difference in preterm delivery between women with a singleton pregnancy or twin pregnancy and GDM. NOTE: Weights are from random-effects model.

Five studies described the difference in having a preterm delivery between singleton GDM and twin GDM pregnancies. The pooled OR was 0.07 (95%CI: 0.06 to 0.09; I^2^ = 49%), indicating that the women with a singleton pregnancy and GDM had a 93% lower risk of preterm delivery when compared to women with a twin pregnancy and GDM (p<0.001) (Fig 6).

#### Respiratory morbidity

**Fig 7:**
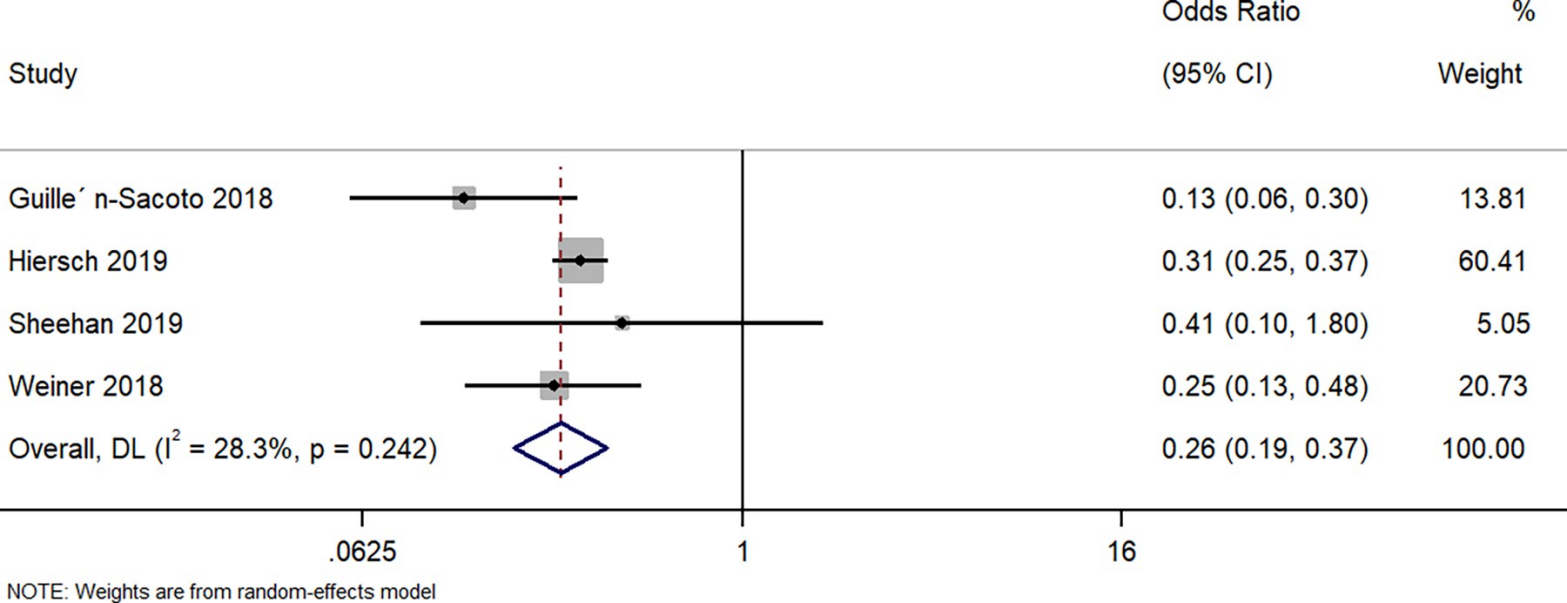
Forest plot showing the difference in respiratory morbidity in neonates between women with a singleton pregnancy or twin pregnancy and GDM. NOTE: Weights are from random-effects model.

Four studies described the difference in respiratory morbidity in neonates born to GDM women with a singleton pregnancy and those born to women with a twin pregnancy and GDM. The pooled OR was 0.26 (95%CI: 0.19 to 0.37; I^2^ = 28.3%), indicating that the neonates born to women with a singleton pregnancy and GDM had a significantly lower (74%) risk of having respiratory morbidity when compared to neonates born to women with a twin pregnancy and GDM (p<0.001) (Fig 7).

#### Hypoglycaemia

**Fig 8:**
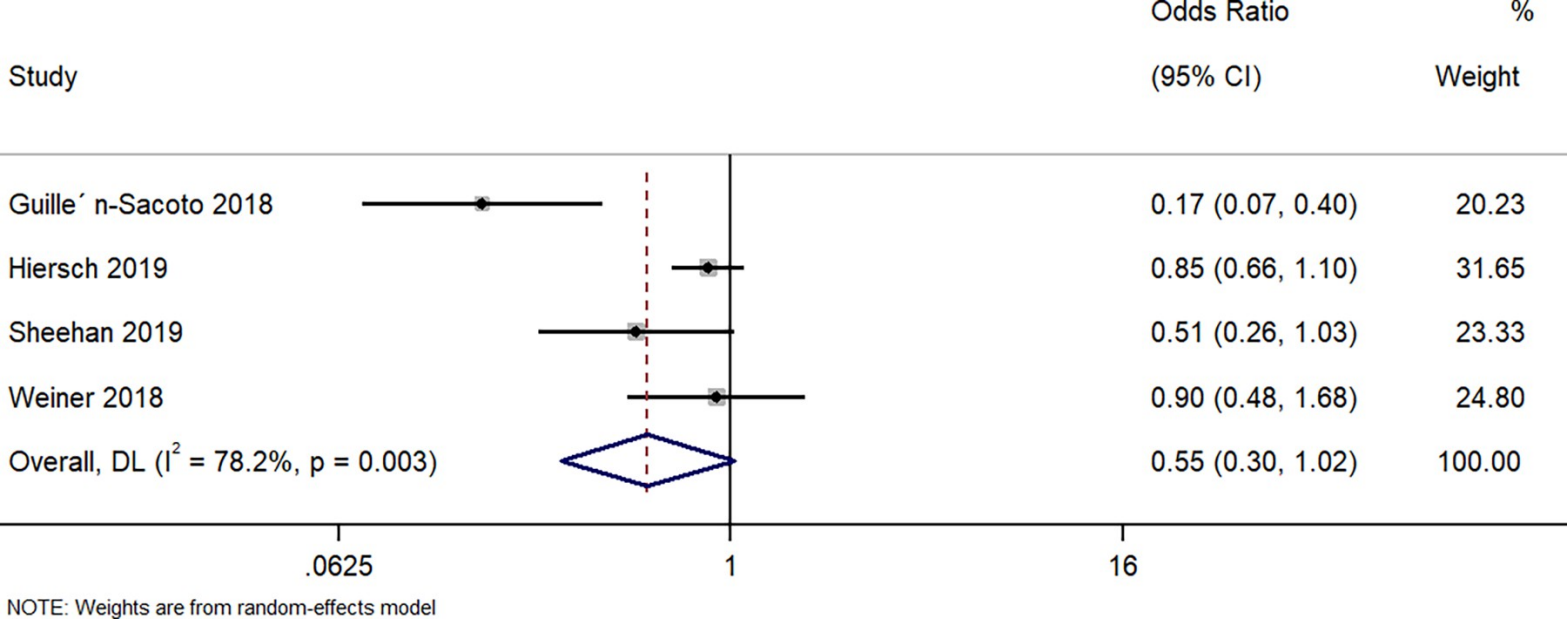
Forest plot showing the difference in neonatal hypoglycaemia between women with a singleton pregnancy or twin pregnancy and GDM. NOTE: Weights are from random-effects model.

Four studies described the difference in hypoglycaemia in neonates between singleton GDM and twin GDM pregnancies. The pooled OR was 0.55 (95%CI: 0.30 to 1.02; I^2^ = 78.2%), indicating that there was no difference in the rate of neonatal hypoglycaemia between both groups (p = 0.06) (Fig 8).

#### Hyperbilirubinemia

**Fig 9:**
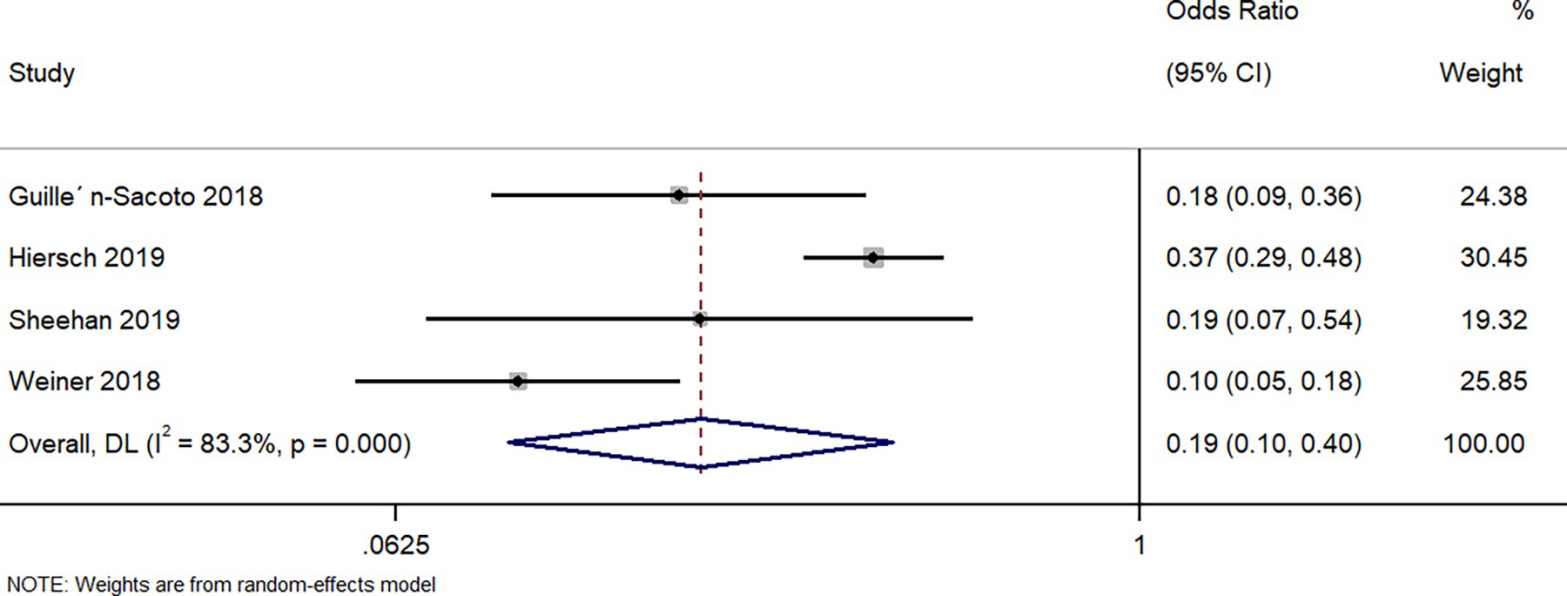
Forest plot showing the difference in neonatal hyperbilirubinemia between women with a singleton pregnancy or twin pregnancy and GDM. NOTE: Weights are from random-effects model.

Four studies described the difference in neonates having hyperbilirubinemia between the neonates born to singleton GDM and those born to twin GDM pregnancies. The pooled OR was 0.19 (95%CI: 0.10 to 0.40; I^2^ = 83.3%), indicating that the neonates born to women with a singleton pregnancy and GDM had a significantly lower (81%) risk of having hyperbilirubinemia when compared to neonates born to women with a twin pregnancy and GDM (p<0.001) (Fig 9).

#### NICU admission

**Fig 10:**
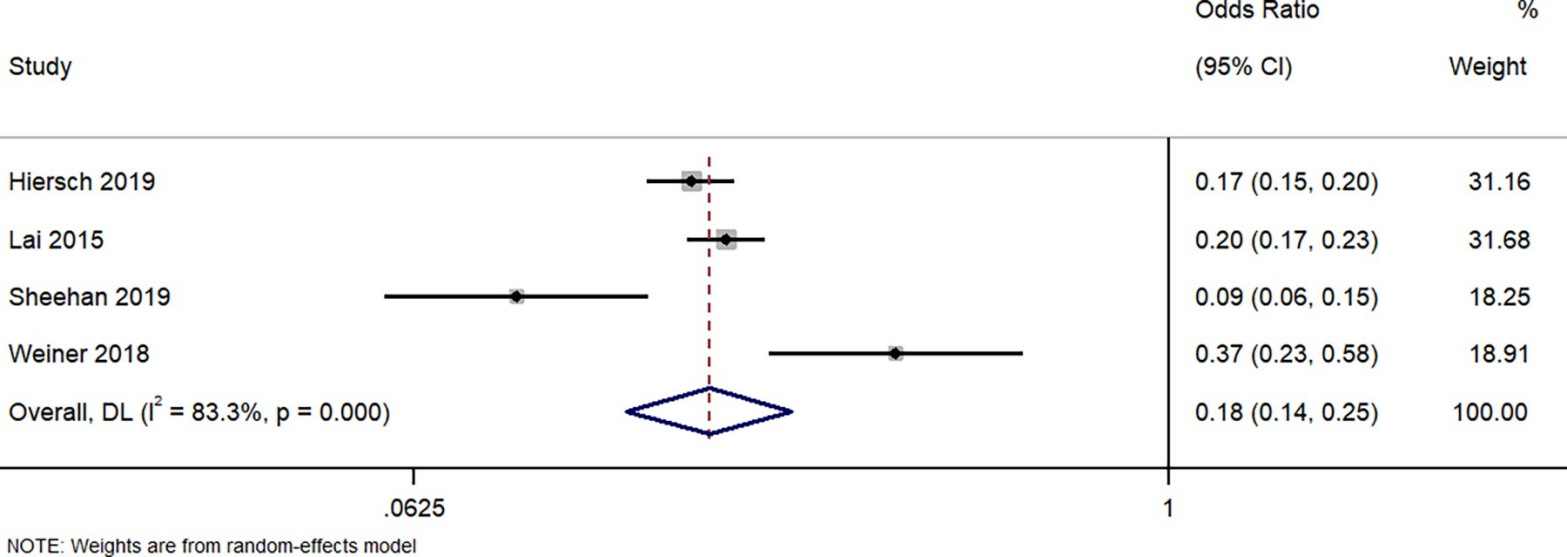
Forest plot showing the difference in NICU admission between women with a singleton pregnancy or twin pregnancy and GDM. NOTE: Weights are from random-effects model.

Four studies have described the difference in neonates requiring NICU admission between the neonates born to singleton GDM and those born to twin GDM pregnancies. The pooled OR was 0.18 (95%CI: 0.14 to 0.25; I^2^ = 83.3%), indicating that the neonates born to women with a singleton pregnancy and GDM have a significantly lower (82%) risk of requiring NICU admission when compared to neonates born to women with a twin pregnancy and GDM (p<0.001) (Fig 10).

### Additional analysis

The sensitivity analysis did not find any appreciable variation in the effect size (in terms of magnitude and direction). This suggests that there was no overall single-study effect on the estimate of any outcome. Due to the small number of papers, a funnel plot for publication bias and meta-regression for examining heterogeneity cannot be performed.

## Discussion

This systematic review and meta-analysis aimed to examine the risk of various maternal and neonatal outcomes in women with a singleton pregnancy and a twin pregnancy that are complicated by GDM. In total, 8 studies fulfilled the eligibility criteria. Most of these studies were conducted in Western countries, like Canada and Spain. While all the studies followed the retrospective design, most of them were of good to a satisfactory quality.

The results presented here highlight the divergent effect of GDM on almost all neonatal outcomes (except neonatal hypoglycaemia), especially the risk of caesarean section. Sensitivity analysis did not reveal any significant single-study effect on the magnitude or direction of the association.

While there were no previous attempts to compare maternal and neonatal outcomes in GDM women with singleton and twin pregnancies, we explored the potential impact of such association using the previous literature. Our results show that women with a singleton pregnancy and GDM had a significantly lower risk of having a caesarean section than women with a twin pregnancy and GDM. These results are surprising given that the growth potential during the third trimester in a twin pregnancy is lower, leading to a reduced possibility of developing macrosomia [23]. However, these results could be due to the inherent indications for caesarean delivery associated with GDM or twin pregnancies, such as a discrepancy in fetal growth, higher risk of placenta previa, malpresentation, and patient and doctor preference [13].

Our study also shows that GDM women with a singleton pregnancy have a significantly lower risk of having a preterm delivery and SGA babies compared to women with a twin pregnancy and GDM. It can be argued that GDM-induced accelerated growth in twins should not be concerning, given that it is unlikely to cause neonatal complications like birth trauma or shoulder dystocia. GDM-induced growth in twins could actually be beneficial, given the slower rate of growth in twins during the third trimester, thus raising the risk of growth restriction [24–27]. These factors are most likely why the impact of GDM in excess foetal growth is counteracted. However, accelerated growth within the foetus may be associated with similar foetal programming as singleton pregnancies with GDM, ultimately leading to long-term metabolic complications including diabetes, obesity, and cardiovascular disease [28–32]. Hence, longitudinal studies conducted over a longer period among the twin GDM infants are necessary to answer this question.

Our results suggest that women with a singleton pregnancy and GDM had a significantly lower risk of delivering neonates with hyperbilirubinemia or respiratory morbidity than women with a twin pregnancy. The mechanism behind such association was unclear and further longitudinal studies are required to identify the possible reason(s) for the association with adverse metabolic neonatal outcomes and twin GDM. We also found a significantly lower NICU admission rate in women with a singleton pregnancy and GDM. We may speculate that GDM women with twin pregnancies have a significantly higher overall risk of adverse neonatal outcomes leading to admission to NICU for closer monitoring and observation till their recovery. An alternative hypothesis could be that twin pregnancies are associated with an overall high risk which may be further increased by maternal GDM. Hence, the neonates might be admitted to the NICU irrespective of the development of complications for close monitoring [9].

The main strength of this review was the accurate methodology and comprehensive literature search. In addition, this review adds to the limited evidence available on the association between singleton & twin pregnancies with GDM and adverse maternal and neonatal outcomes. We found that the majority of the included studies were of better quality and had little to no outcome heterogeneity. This raises the evidence’s credibility and the findings’ external validity (generalizability). Sensitivity analysis revealed no discernible alteration in the magnitude or direction of study estimate.

However, there are some limitations to this study. For some of the outcomes, significant between-study variability was found. It is difficult to explore such significant heterogeneity by meta-regression because there are so few papers. Due to similar factors, we were unable to evaluate the probability of publication bias with any of the results. Since almost all of the investigations were retrospective, it was difficult to determine any causal relationships. The determination of the proper effect size and the development of evidence-based recommendations for the hospital context require further longitudinal research.

Despite the listed limitations, this meta-analysis has specific implications for the clinical practice. Women with twin pregnancy and GDM should be classified as a specific target group for providing focussed care during their antenatal period. These patients require more stringent follow-ups and high level of clinical care throughout their antenatal, natal and postnatal period. The neonates born out of these women also requires intensive targeted care throughout their neonatal period and intermittent follow-ups till they pass the infancy period.

## Conclusions

The results presented here highlight how maternal outcomes like caesarean section and neonatal outcomes like SGA neonates, preterm delivery, respiratory morbidity, hyperbilirubinemia, and NICU admission are significantly greater in women with twin pregnancies and GDM. While several outcomes were similar between the two groups, a thorough understanding of the intervention strategies for women with twin pregnancies who are diagnosed with GDM is of great importance. Further, it is paramount that clinicians are aware of the significantly increased risk in GDM women with a twin pregnancy.

## Supporting information

S1 Appendix Search strategy. (PDF)
