# Supplementary material for: Maternal and neonatal outcomes of singleton versus twin pregnancies complicated by gestational diabetes mellitus: A systematic review and meta-analysis
Source: PLoS One. 2023 Jan 25;18(1):e0280754. doi: 10.1371/journal.pone.0280754 (PMC9876238; doi:10.1371/journal.pone.0280754)
Supplement: S1 Appendix — (PDF) [file pone.0280754.s001.pdf]

EMBASE:

('pregnancy diabetes mellitus'/exp OR 'diabetes mellitus gravidarum' OR 'diabetes, gestational' OR 'diabetes, pregnancy' OR 'gestational diabetes' OR 'gestational diabetes mellitus' OR 'pregnancy diabetes' OR 'pregnancy diabetes mellitus' OR 'pregnancy in diabetics') AND 'singleton pregnancy'/exp AND ('twin pregnancy'/exp OR 'pregnancy, twin' OR 'twin pregnancy' OR 'twinning rate') – 15 results

PubMed:

"diabetes, gestational"[MeSH Terms] AND "pregnancy, twin"[MeSH Terms] AND (("singleton"[All Fields] OR "singletons"[All Fields]) AND ("pregnancy"[MeSH Terms] OR "pregnancy"[All Fields] OR "pregnancies"[All Fields] OR "pregnancy s"[All Fields]))

Translations

Singleton: "singleton"[All Fields] OR "singletons"[All Fields]

Pregnancy: "pregnancy"[MeSH Terms] OR "pregnancy"[All Fields] OR "pregnancies"[All Fields] OR "pregnancy's"[All Fields]
